# Molecular mechanisms for the modulation of blood pressure and potassium homeostasis by the distal convoluted tubule

**DOI:** 10.15252/emmm.202114273

**Published:** 2021-12-20

**Authors:** María Castañeda‐Bueno, David H Ellison, Gerardo Gamba

**Affiliations:** ^1^ Department of Nephrology and Mineral Metabolism Instituto Nacional de Ciencias Médicas y Nutrición Salvador Zubirán Tlalpan, Mexico City Mexico; ^2^ Division of Nephrology and Hypertension Department of Medicine Oregon Health and Science University Portland OR USA; ^3^ Oregon Clinical & Translational Research Institute Oregon Health & Science University Portland OR USA; ^4^ VA Portland Health Care System Portland OR USA; ^5^ Molecular Physiology Unit Instituto de Investigaciones Biomédicas Universidad Nacional Autónoma de México Tlalpan, Mexico City Mexico

**Keywords:** epithelial transport, familial hyperkalemic hypertension, gitelman syndrome, potassium, SESAME/EAST syndrome, Cardiovascular System, Urogenital System

## Abstract

Epidemiological and clinical observations have shown that potassium ingestion is inversely correlated with arterial hypertension prevalence and cardiovascular mortality. The higher the dietary potassium, the lower the blood pressure and mortality. This phenomenon is explained, at least in part, by the interaction between salt reabsorption in the distal convoluted tubule (DCT) and potassium secretion in the connecting tubule/collecting duct of the mammalian nephron: In order to achieve adequate K^+^ secretion levels under certain conditions, salt reabsorption in the DCT must be reduced. Because salt handling by the kidney constitutes the basis for the long‐term regulation of blood pressure, losing salt prevents hypertension. Here, we discuss how the study of inherited diseases in which salt reabsorption in the DCT is affected has revealed the molecular players, including membrane transporters and channels, kinases, and ubiquitin ligases that form the potassium sensing mechanism of the DCT and the processes through which the consequent adjustments in salt reabsorption are achieved.

## Graphical abstract

In this review, G. Gamba *et al* present the current knowledge on the regulation of sodium and potassium homeostasis in the distal convoluted tubule, and the related control of blood pressure.

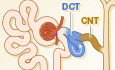

**Glossary**
Hyperkalemia: Levels of plasma [K+] that are above the normal physiological range
Macula densa: A group of specialized epithelial cells that share morphological and functional features with the cells of the thick ascending limb of Henle’s loop (TAL). These cells are localized in the transition between the TAL and the DCT, right where the tubule contacts the glomerulus after having descended to and ascended from the renal medulla. They are equipped with the molecular machinery that allows them to sense the delivery of NaCl to this nephron segment and respond accordingly to modulate the glomerular filtration rate
Natriuresis: The excretion of Na+ in the urine
Nephron: Functional unit of the kidney that is composed of a glomerulus, a bunch of capillaries surrounded by a capsule of epithelial cells to which the plasma is filtered, and a tubule, formed by a monolayer of epithelial cells that modify the filtered plasma to produce urine through the processes of reabsorption and secretion
Pressure natriuresis: Mechanism involved in the long‐term regulation of blood pressure. Any shifts in blood pressure sensed by the kidneys result in an adjustment of water and salt urinary output that normalizes blood pressure

## Introduction

**Figure 1:**
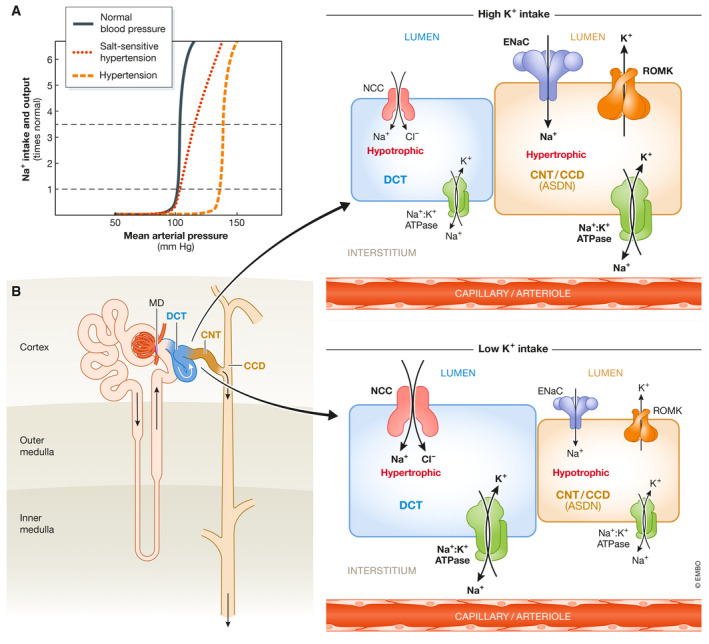
The renal pressure natriuresis mechanism has a central role in long‐term blood pressure regulation (A) Any shifts in blood pressure sensed by the kidneys result in an adjustment of water and salt urinary output that normalizes blood pressure (black line). In hypertensive patients, the kidneys reabsorb more salt at a given blood pressure value, and thus, salt balance is achieved by increasing blood pressure (orange line). In salt‐sensitive hypertension, the slope of the curve is modified, and thus, changes in salt intake have a notable impact on blood pressure levels (red line). This is observed, for example, when one mechanism for regulation of renal salt excretion is lost, as in primary aldosteronism (Adapted from Guyton AC, Coleman TG, Young DB, *et al*: Salt balance and long‐term blood pressure control. Annu Rev Med 31:15, 1980). (B) Right after the macula densa (MD), the first portion of the distal nephron (depicted in the inset) is where the fine tuning of salt and K^+^ urinary excretion takes place. It is composed by three functionally distinct segments: the distal convoluted tubule (DCT), followed by the connecting tubule (CNT), and the cortical collecting duct (CCD). Here, apical Na^+^ uptake is driven by the Na^+^ gradient generated by the basolateral Na^+^/K^+^ ATPase (in green). In the DCT, NCC (in red) is the major apical Na^+^ entry pathway. In the CNT and CCD, Na^+^ crosses the apical membrane via the Na^+^ channel ENaC (blue). ENaC activity generates a lumen‐negative electrical gradient that drives K^+^ efflux through apical ROMK channels (yellow). Thus, ROMK‐mediated K^+^ secretion is dependent on ENaC activity. This K^+^ secretory system is modulated by aldosterone; hence, these segments (CNT and CCD) constitute the aldosterone sensitive distal nephron (ASDN). In addition, in intercalated cells present within the CNT and CCD segments (not depicted here) flow‐activated BK channels can mediate K^+^ secretion under high luminal flow conditions. Although no net K^+^ reabsorption or secretion occurs in the DCT, NCC activity can modulate K^+^ secretion in the ASDN by modulating Na^+^ delivery. Thus, NCC activity is modulated in response to changes in dietary K^+^ intake that indirectly affect K^+^ secretion. Moreover, changes in NCC activity promote the remodeling of distal nephron segments. For instance, high NCC activity is associated with DCT hypertrophy and CNT/CCD hypotrophy (Grimm *et al*, 2017), whereas low NCC activity is associated with DCT hypotrophy (Loffing *et al*, 1996; Schnoz *et al*, 2020). Created with Biorender.com.

Arterial pressure is modulated in seconds to hours by primary signaling systems such as neurotransmitters and vasoactive hormones that keep it within narrow limits. However, the long‐term regulation of blood pressure is primarily achieved by a renal mechanism that Arthur C. Guyton denominated pressure natriuresis (Fig 1A).

Communication between intrarenal blood vessels and tubules modulates salt and water reabsorption, and thus the intravascular filling pressure. If blood pressure is increased by any mechanism, pressure natriuresis will increase salt excretion in the urine, extracellular fluid volume will be decreased, and consequently blood pressure will be reduced. In chronic hypertension, salt reabsorption by the kidneys is pathologically increased, which results in volume expansion, and increased blood pressure. High blood pressure promotes natriuresis that results in restoration of salt balance at the expense of hypertension. In other words, arterial hypertension is the pathophysiological response to very slight but continuous chronic salt retention (Fig 1). This is a very slow mechanism. Even in patients with an inherited monogenic cause of hypertension, the increase in blood pressure takes years to develop.

Several observations support the concept that the kidneys are in charge of the long‐term regulation of blood pressure. In animal models, attempts to increase blood pressure by any means are unsuccessful if pressure natriuresis is allowed to preform properly (Guyton, 1991). Renal transplantation between genetically normotensive or hypertensive rodents has shown that hypertension follows the kidney (Ivy & Bailey, 2014). Renal transplantation in primary hypertensive patients who developed end stage renal disease due to hypertensive nephropathy cured not only the renal failure but also the hypertension (Curtis *et al*, 1983). Finally, nearly all inherited monogenic diseases featuring chronic changes in blood pressure are due to mutations in genes that encode for renal salt transport proteins or their regulators.

In the past few years, major advances have been made in the understanding of a complex regulatory system that operates in the distal convoluted tubule of the nephron to modulate salt and potassium homeostasis and thus arterial blood pressure. These advances will be discussed here.

## Renal salt and potassium transport in the distal nephron

The nephron is divided into proximal and distal by the macula densa (Fig 1B). Any change in salt reabsorption in the proximal nephron is compensated by the tubuloglomerular feedback, a mechanism that regulates glomerular filtration in response to the amount of NaCl delivered to the macula densa. In contrast, distal nephron function is not subjected to this regulation, and thus, changes in salt reabsorption occurring here are reflected in the final urine. Thus, the NaCl cotransporter (NCC), located in the distal convoluted tubule (DCT), just after the macula densa, is well suited to control urinary Na excretion and hence blood pressure.

NCC is present in the apical membrane of the DCT. This is the target of thiazide‐type diuretics that are among the primary drugs prescribed for the treatment of hypertension. The subsequent segments to the DCT are the connecting tubule (CNT) and the collecting ducts (CD). Each CD receive fluid from five to ten CNTs. The apical membranes of CNTs and CDs express the Na^+^ and K^+^ channels ENaC (Epithelial Na^+^ channel) and ROMK (Renal Outer Medullary K^+^ channel), respectively (Fig 1B). These are the segments where regulation of urinary K^+^ excretion takes place. Na^+^ reabsorption by ENaC is electrogenic, producing a voltage negative lumen in the CNT/CD that is the driving force for K^+^ secretion through ROMK and the BK channels. K^+^ secretion is modulated by multiple signals that include the amount of Na^+^ and fluid delivered to the CNT/CD. Thus, changes in NCC function in the previous segment can influence the rate of K^+^ secretion in the CNT/CD by affecting Na^+^ and fluid delivery (Fig 1B) (Yang *et al*, 2021). DCT is divided in two segments: DCT1 that exclusively express NCC in the apical membrane and DCT2 that is a transition segment between the DCT1 and the CNT/CD. DCT2 apical membrane contains NCC, ENaC, and ROMK.

It is known that dietary K^+^ modulates blood pressure. The higher the K^+^ ingestion, the lower the blood pressure and vice versa (Mente *et al*, 2014) (Sacks *et al*, 2001; Ma *et al*, 2021). This has been observed in open population and in several potassium supplementation trials. However, a recent meta‐analysis of 32 trials, in which potassium supplementation was tested in hypertensive patients (Filippini *et al*, 2020), revealed that the blood pressure lowering effect is lost when the amount of supplemented potassium is above 100 mmol/day. Surprisingly, with these supplementation levels, blood pressure actually increases, suggesting that in patients with hypertension, high levels of K^+^ ingestion may exert other effects on the cardiovascular system.

Plasma K^+^ must always be maintained within a very narrow range (3.5–5.5 mEq/l), whereas the body can easily adjust to mild changes in volume status. Thus, K^+^ balance is prioritized over salt balance and high K^+^ ingestion reduces salt reabsorption in the DCT, to increase Na^+^ delivery to the CNT/CD, promoting K^+^ secretion, at the expense of reducing blood pressure. The contrary occurs with low K^+^ ingestion.

## The study of inherited diseases revealed a molecular mechanism explaining the inverse relationship between K^+^ intake and blood pressure levels

Inactivating mutations in the *SLC12A3* gene that encodes NCC cause Gitelman’s disease that features salt wasting with low blood pressure and hypokalemic, metabolic alkalosis (Simon *et al*, 1996). Additionally, inactivating mutations in the *KCNJ10* gene that encodes the Kir4.1 K^+^ channel, present in the basolateral membrane of the DCT, causes a complex neurological disease that also features a Gitelman‐like phenotype (Scholl *et al*, 2009) (Bockenhauer *et al*, 2009). In contrast, mutations in four genes produce a disease with a mirror phenotype: inherited salt‐sensitive hypertension with hyperkalemia and metabolic acidosis, known as Familiar Hyperkalemic Hypertension (FHHt) or pseudohypoaldosteronism type II, that is mainly due to NCC overactivation (Murillo‐de‐Ozores *et al*, 2020). Two of these genes encode for the With No lysine (K) kinases WNK1 and WNK4 (Wilson *et al*, 2001) and the other two encode for proteins known as Kelch like 3 (KLHL3) and Cullin 3 (CUL3) that are part of a RING‐type ubiquitin ligase complex (Boyden *et al*, 2012; Louis‐Dit‐Picard *et al*, 2012). These human diseases hinted that the link between blood pressure and potassium regulation resides, at least in part in the DCT, because reduced activity of DCT results in hypotension and hypokalemia, whereas increased activity of DCT causes hypertension and hyperkalemia.

## The proteins whose altered function cause the FHHt and SeSAME/EAST syndromes regulate the activity of NCC

**Figure 2:**
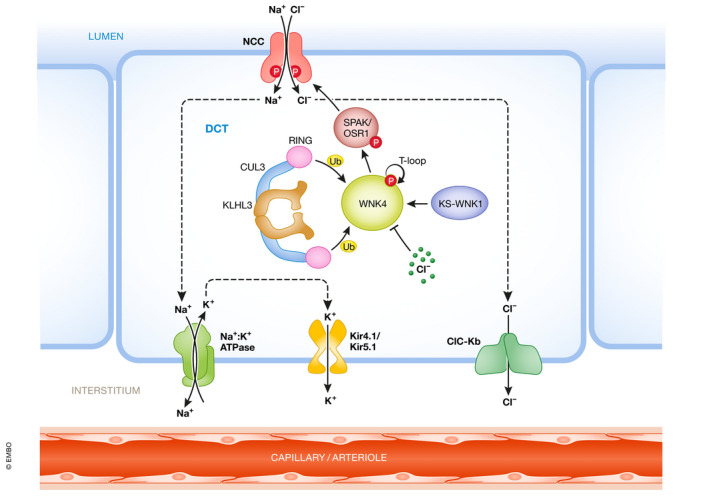
Key molecular players in DCT cells NaCl reabsorption in the DCT occurs through the concerted action of NCC in the apical membrane and the Na^+^/K^+^ ATPase, the ClC‐Kb channels, and the Kir4.1/5.1 K^+^ channels in the basolateral side. NCC phosphorylation is modulated by the WNK4‐SPAK/OSR1 pathway, which is modulated by intracellular chloride concentration and the activity of the CUL3‐KLHL3 ubiquitin ligase complex. The kidney‐specific, catalytically inactive, short isoform of WNK1 (KS‐WNK1) can bind WNK4 and promote its activation under certain conditions. Created with Biorender.com.

NCC is a twelve transmembrane spanning protein that belongs to the electroneutral cation‐coupled chloride cotransporters family (SLC12) (Gamba, 2005). In the kidney, it is exclusively present in the apical membrane of DCT cells, where it participates in salt reabsorption (Fig 2). Its activity is modulated by phosphorylation of key residues in the amino terminal domain. When threonine residues 55 and 60, and serine 73 of hNCC (Thr 53, 58, and 71 in mouse and rat NCC) are phosphorylated, the activity of the cotransporter is increased (Pacheco‐Alvarez *et al*, 2006; Richardson *et al*, 2008). Threonine 60 is the master site for its regulation. The use of phosphoantibodies recognizing these sites has been of remarkable help for the study of NCC activity in a diversity of mouse models (Hadchouel *et al*, 2016; Ostrosky‐Frid *et al*, 2019).

The kinases responsible for NCC phosphorylation are the STE20‐Proline Alanine rich Kinase (SPAK) and the Oxidative Stress Responsive kinase 1 (OSR1) (Piechotta *et al*, 2002; Dowd & Forbush, 2003; Vitari *et al*, 2005; Richardson *et al*, 2008) (Fig 2). NCC possesses a SPAK binding site near the phosphorylation sites that allows interaction with, and phosphorylation by, these kinases. In the absence of phosphorylation, the activity of NCC is completely absent (Pacheco‐Alvarez *et al*, 2006; Yang *et al*, 2013).

SPAK and OSR1 are targets of the WNK kinases. To be active and promote the NCC phosphorylation, SPAK/OSR1 must be phosphorylated in key residues by the WNK kinases (Fig 2) (Vitari *et al*, 2005, 2006). The most important sites for activation are threonine residues 243 and 185 in mSPAK and mOSR1, respectively. Elimination of these sites in vitro renders them catalytically inactive (Vitari *et al*, 2005). Elimination of SPAK’s T243 in mice produces a Gitelman‐like syndrome due to the reduction of NCC activity (Rafiqi *et al*, 2010). Serine 383 in mSPAK and serine 325 in mOSR1 are also phosphorylated by WNKs. Although the role of these sites for kinase activation is more controversial (Vitari *et al*, 2005; Gagnon & Delpire, 2010), their phosphorylation has been extensively used to assess the kinases’ activation state using phosphoantibodies. In mouse, constitutive activation of SPAK, exclusively in the DCT1, recapitulates the full phenotype of FHHt, strongly suggesting that activation of NCC alone is enough to produce the disease phenotype (Grimm *et al*, 2017).

There are four WNK kinases genes in mammals known as WNK1 to WNK4 (Verissimo & Jordan, 2001). WNK2 is almost exclusively expressed in the central nervous system and has been identified as a tumor suppressor gene (Rinehart *et al*, 2011). WNK3 is present in many cells and tissues and has been shown to be involved in cell volume regulation due to its effects on SLC12 transporters (Cruz‐Rangel *et al*, 2012; Pacheco‐Alvarez *et al*, 2020). Mutations in WNK3 result in a very complex neurological disease known as Anderman’s syndrome (Howard *et al*, 2002). WNK1 and WNK4 are also expressed in many cells, but when mutated they produce FHHt whose phenotype is exclusively the consequence of DCT malfunction. Another disease due to mutations in a WNK1 exon that is exclusively expressed in neurons is the Hereditary Sensory Neuropathy type II (Shekarabi *et al*, 2008).

The With No lysine (K) kinases (WNKs) owe their name to the atypical positioning of the catalytic lysine in subdomain I instead of in subdomain II of the kinase domain where it is found in all other serine/threonine kinases (Xu *et al*, 2000). In the kidney, the WNK1 gene gives rise to two isoforms due to the presence of an alternative promoter located in intron 4 (Delaloy *et al*, 2003; O'Reilly *et al*, 2003). The long WNK1 (L‐WNK1), whose transcription starts in exon 1, contains the kinase domain and is expressed at low levels in the kidney. The short WNK1, known as KS‐WNK1 due to its kidney‐specific expression, contains a unique N‐terminal segment that is 30 residues long and is encoded in exon 4a, followed by the rest of the protein starting at the polypeptide sequence encoded by exon 5. KS‐WNK1 thus contains no kinase domain. This isoform’s transcript is highly expressed in the DCT and is almost exclusively present in this part of the nephron (Vidal‐Petiot *et al*, 2012). The role of KS‐WNK1 in DCT physiology is still debated as reviewed below. Large intronic deletions (20 to 40 kilobases long) occurring within the first intron of WNK1 produce FHHt, most likely as a consequence of the ectopic expression of L‐WNK1 in the DCT.

The WNK4 kinase is highly expressed in the DCT where it is the main regulatory kinase of the SPAK‐NCC pathway (Castaneda‐Bueno *et al*, 2012; Murillo‐de‐Ozores *et al*, 2021). All WNKs contain a highly conserved acidic region located in the first portion of the C‐terminal regulatory domain (EPEEP**E**A**DQ**H in WNK4, bold letters mark the positions in which point mutations have been identified in FHHt kindreds). Missense mutations within this motif in WNK4 result in FHHt (Wilson *et al*, 2001). The dominant pattern of inheritance of the disease caused by these mutations suggested that they result in a gain of function. As shown in Fig 2, it is now accepted that WNK4 activates the SPAK‐NCC pathway. However, the field was confused for years because initial evidence from in vitro and in vivo models suggested that WNK4 could inhibit (Wilson *et al*, 2003; Yang *et al*, 2003; Cai *et al*, 2006; Lalioti *et al*, 2006; Mu *et al*, 2011) or activate (Na *et al*, 2012; Wakabayashi *et al*, 2013) NCC. The first clear evidence that WNK4 activates NCC was the observation that elimination of WNK4 in mice resulted in a remarkable decrease in NCC expression and phosphorylation and the consequent Gitelman‐like phenotype (Castaneda‐Bueno *et al*, 2012). Later, it was shown that the confusion was due to the fact that WNK4 activity is sensitive to intracellular chloride concentration ([Cl^−^]_i_). First, Piala *et al* (2014), through the structural analysis of WNK1’s kinase domain, identified a chloride binding site within the active site of WNK1 that, when occupied, prevents the autophosphorylation and thus activity of the kinase. Then, Bazua‐Valenti *et al* (2015) demonstrated that the same chloride binding site is present in WNK4 that, when eliminated by the L322F substitution (in hWNK4), renders WNK4 constitutively active, so that it can no longer be inhibited by Cl^−^. In this regard, it was previously shown that NCC activity is modulated by [Cl^−^]_i_ through phosphorylation of the cotransporter (Pacheco‐Alvarez *et al*, 2006). Therefore, as shown in Fig 2, the activity of WNK4 in the DCT is modulated by [Cl^−^]_i_. If [Cl^−^]_i_ declines, WNK4 is autophosphorylated acquiring the capacity to activate SPAK and OSR1 that in turn activate NCC. In contrast, increased [Cl^−^]_i_ prevents WNK4 activity and, thus, SPAK/OSR1 and NCC activation.

WNK4 is perfectly suited for its regulatory role of NCC activity in the DCT given that it is the WNK kinase with the highest sensitivity to inhibition by Cl^−^ (Terker *et al*, 2016b; Pacheco‐Alvarez *et al*, 2020) and the assessment of [Cl^−^]_i_ in DCT cells by different groups has shown that it is lower than in most mammalian cells (Boettger *et al*, 2002; Weinstein, 2005; Su *et al*, 2020). Because of their lower sensitivity to Cl^−^, if L‐WNK1 and WNK3 were expressed in the DCT, they would not be expected to be inhibited by the low Cl^−^ environment of these cells and, thus, would keep NCC constantly active. Therefore, it makes sense that these kinases are not expressed in the DCT under physiological conditions (Lee *et al*, 2015; Chen *et al*, 2021b). Instead, disease‐causing deletions occurring within intron one of *WNK1* promote the ectopic expression of L‐WNK1 (like in WNK1^+/FHHt^ mice) in the DCT and produce FHHt (Vidal‐Petiot *et al*, 2013; Chavez‐Canales *et al*, 2014). In addition, elimination of WNK4 in mice that also contain the KLHL3‐R528H mutation, which alone causes a severe FHHt phenotype, results in almost complete absence of NCC activity with a Gitelman‐like phenotype (Susa *et al*, 2017). This contrasts with the observation that in WNK1^+/FHHt^ mice that present the ectopic presence of L‐WNK1 in the DCT, additionally knocking out WNK4 does not correct the FHHt phenotype, suggesting that DCT L‐WNK1 expression in these mice can compensate for the absence of WNK4 (Chavez‐Canales *et al*, 2014).

When mutations in *WNK1* and *WNK4* were found to cause FHHt (Wilson *et al*, 2001), it was noticed that most affected families had no mutations in these genes, but the genome linkage analysis had no power to detect other involved genes. Ten years later, when whole exome sequencing became possible, FHHt‐causative mutations in the *CUL3* and *KLHL3* genes were identified. CUL3 and KLHL3 are part of a RING‐type ubiquitin ligase complex (Boyden *et al*, 2012; Louis‐Dit‐Picard *et al*, 2012). *KLHL3* mutations can be dominant or recessive, whereas *CUL3* mutations are dominant and mostly *de novo* (Ostrosky‐Frid *et al*, 2020). Analysis of the age at which hypertension develops, the percentage of family members with the mutation that also present hypertension, and the levels of serum K^+^, HCO_3_^−^, and pH (see Table 1), showed that the severity of the phenotype is related to the mutated gene with the following profile (from more to less severe): CUL3 > KLHL3 dominant > KLHL3 recessive > WNK4 > WNK1. It was proposed that CUL3 and KLHL3 must lie upstream of WNK kinases and this was quickly demonstrated when it was shown that the CUL3‐KLHL3 ubiquitin ligase complex binds and ubiquitylate WNKs, marking them for proteasomal degradation (Ohta *et al*, 2013; Shibata *et al*, 2013; Wakabayashi *et al*, 2013). CUL3 forms a dimer that in turn binds to a KLHL3 dimer through the BTB domain of KLHL3. KLHL3 interacts with WNK kinases through its KLHL propeller domain (Fig 2). The KLHL3 binding site in WNKs is the highly conserved acidic motif (see above). Mutations in the acidic motif of WNK4 that produce FHHt abrogate the recognition of the kinase by the CUL3‐KLHL3 E3 complex.

**Table 1:** Genetic diseases with altered DCT function.

Disease	Affected gene	Affected protein	Mutation	Mendelian inheritance	Proposed mechanism	
Gitelman syndrome	*SLC12A3*	NCC	Loss‐of‐function missense, nonsense, or frameshift‐introducing mutations in > 100 different positions along the protein, small deletions, mutations at donor and acceptor splice sites, etc.	Autosomal recessive	Impaired NCC activity due to impaired protein synthesis, increased cotransporter degradation, impaired trafficking to plasma membrane, impaired cotransporter function, etc.	Acuna *et al* (2011), Gamba (2005), Sabath *et al* (2004)
FHHt	*WNK1*	WNK1	Deletions in intron 1	Autosomal dominant	Ectopic L‐WNK1 expression in the DCT	Vidal‐Petiot *et al* (2013), Wilson *et al* (2001)
		WNK1	Missense mutations in the acidic domain	Autosomal dominant	Decreased KS‐WNK1 degradation in the DCT	Louis‐Dit‐Picard *et al* (2020)
	*WNK4*	WNK4	Missense mutations in the acidic domain	Autosomal dominant	Decreased WNK4 degradation in the DCT	Brooks *et al* (2012), Golbang *et al* (2005), Gong *et al* (2008), Shibata *et al* (2013), Wakabayashi *et al* (2013), Wilson *et al* (2001)
		WNK4	Missense mutations in the C‐terminal regulatory region: R1185C and K1169E	Autosomal dominant	Disruption of inhibitory domain that promotes increased WNK4 activity	Na *et al* (2012), Wilson *et al* (2001), Zhang *et al* (2011)
	*KLHL3*	KLHL3	Several missense mutations clustered in the BTB domain and specific regions of the kelch propeller domain	Autosomal dominant	Decreased WNK4 and KS‐WNK1 degradation in the DCT	Boyden *et al* (2012), Louis‐Dit‐Picard *et al* (2012), Susa *et al* (2014), Susa *et al* (2017)
		KLHL3	Several loss‐of‐function missense, nonsense, and splicing‐altering mutations	Autosomal recessive	Decreased WNK4 and KS‐WNK1 degradation in the DCT	Boyden *et al* (2012), Louis‐Dit‐Picard *et al* (2012), Sasaki *et al* (2017)
	*CUL3*	CUL3	Mutations that affect the splicing of exon 9 and result in an internal deletion of 57 amino acid residues in the protein	Autosomal dominant	Decreased WNK4 and KS‐WNK1 degradation in the DCT; impaired vascular relaxation through activation of RhoA‐ROCK pathway	Abdel Khalek *et al* (2019), Boyden *et al* (2012), Ferdaus *et al* (2017), Ostrosky‐Frid *et al* (2020)
SESAME / EAST syndrome	*KCNJ10*	Kir4.1	Loss‐of‐function missense or nonsense mutations	Autosomal recessive	Impaired function of Kir4.1/Kir5.1 heterotetramers in the DCT leading to decreased basolateral K^+^ conductance	Bockenhauer *et al* (2009), Scholl *et al* (2009), (Reichold *et al* (2010)

All cases of FHHt caused by mutations in the *CUL3* gene are due to the skipping of exon 9 as a result of aberrant splicing. The resulting protein is 57 amino acid residues shorter (Ostrosky‐Frid *et al*, 2020). Complexes containing CUL3‐Δ9 apparently promote KLHL3 ubiquitylation (Cornelius *et al*, 2018). Thus, the Δ9 mutations are gain of function mutations that promote KLHL3 degradation, attenuating WNK4 ubiquitination and increasing the half‐life of this kinase. FHHt‐causative mutations in KLHL3 can be dominant or recessive. Mutations affecting the BTB domain (and thus, interaction with CUL3) are dominant, and some mutations affecting the substrate binding propeller domain are dominant as well. Recessive mutations lie in the Back domain (which links the BTB and the propeller domains) and in some regions of the propeller domain (Boyden *et al*, 2012). The difference in the mechanism of disease between the dominant and recessive is not completely understood.

## Role of basolateral channels in the regulation of NCC activity

The basolateral membrane of DCT cells also plays an important role in the regulation of NCC. The Kir4.1 channel is present in this membrane. When the *KCNJ10* gene that encodes this channel is mutated in humans, it produces an inherited disease known as EAST/SeSAME syndrome that features Seizures, Sensorineural deafness, Ataxia, Mental retardation, and Electrolyte disturbances (Bockenhauer *et al*, 2009; Scholl *et al*, 2009). This last component is a salt wasting phenotype with hypokalemia similar to the one observed in Gitelman syndrome. Mice null for Kir4.1 reproduce the disease (Zhang *et al*, 2014). In the DCT, Kir4.1 forms heterodimers with Kir5.1 encoded by the *KNJC16* gene. It was recently demonstrated that some individuals with *KNJC16* disrupting mutations also develop a tubulopathy with hypokalemia and sensory deafness (Schlingmann *et al*, 2021). Thus, inactivation of NCC in the apical membrane or Kir4.1/5.1 channels in the basolateral membrane of the DCT results in similar phenotypes with salt wasting and hypokalemia. It turned out that potassium movement through Kir4.1/5.1 activity modulates NCC activity. As will be detailed in the following section, this is mainly due to the effect that basolateral K^+^ movement exert on basolateral Cl^−^ fluxes. Within the basolateral membrane of DCT cells, the ClC‐Kb channel is expressed. Given that this channel is also expressed in the basolateral membrane of cells of the thick ascending limb of Henle’s loop (TAL), where it plays a key role for basolateral Cl^−^ extrusion, mutations in the gene encoding for ClC‐Kb cause Bartter syndrome type III (Simon *et al*, 1996). Bartter syndrome is a tubulopathy caused by the malfunctioning of Na^+^ reabsorption in the TAL that leads to severe urinary salt wasting, hypokalemia, metabolic alkalosis, hypomagnesemia, and hypercalciuria. Type III Bartter patients, however, typically do not present hypercalciuria, and it is though that this is mainly because in these patients not only Na^+^ reabsorption in the TAL is affected, but also Na^+^ reabsorption in the DCT (Matsunoshita *et al*, 2016; Hennings *et al*, 2017). Given that decreased DCT Na^+^ reabsorption is associated with increased renal Ca^2+^ reabsorption, it has been suggested that this defect counterbalances the decreased Ca^2+^ reabsorption occurring in the TAL.

## Regulation of NCC activity in response to changes in extracellular [K^+^]

NCC activity (measured as phosphorylation) is modulated in response to changes in dietary K^+^ content (Vallon *et al*, 2009; Sorensen *et al*, 2013; Castaneda‐Bueno *et al*, 2014; Terker *et al*, 2015a). Low K^+^ intake promotes NCC activation, whereas high K^+^ intake promotes NCC inhibition. Extreme amounts of K^+^ content have been generally used in the rodent studies, in which these observations have been made (low K^+^ ≤ 0.1%; high K^+^ ≥ 5%)), although Terker *et al* did test diets with various K^+^ contents and observed a dose‐dependent effect of K^+^ ingestion on extracellular K^+^ concentration ([K^+^]_e_) and an inverse linear correlation between [K^+^]_e_ and pNCC levels. Observations by several groups have suggested that this modulation occurs in response to changes in extracellular K^+^ concentration ([K^+^]_e_) (Kim *et al*, 1998; Rengarajan *et al*, 2014; Terker *et al*, 2016a, 2016b; Wolley *et al*, 2017; Boscardin *et al*, 2018).

**Figure 3:**
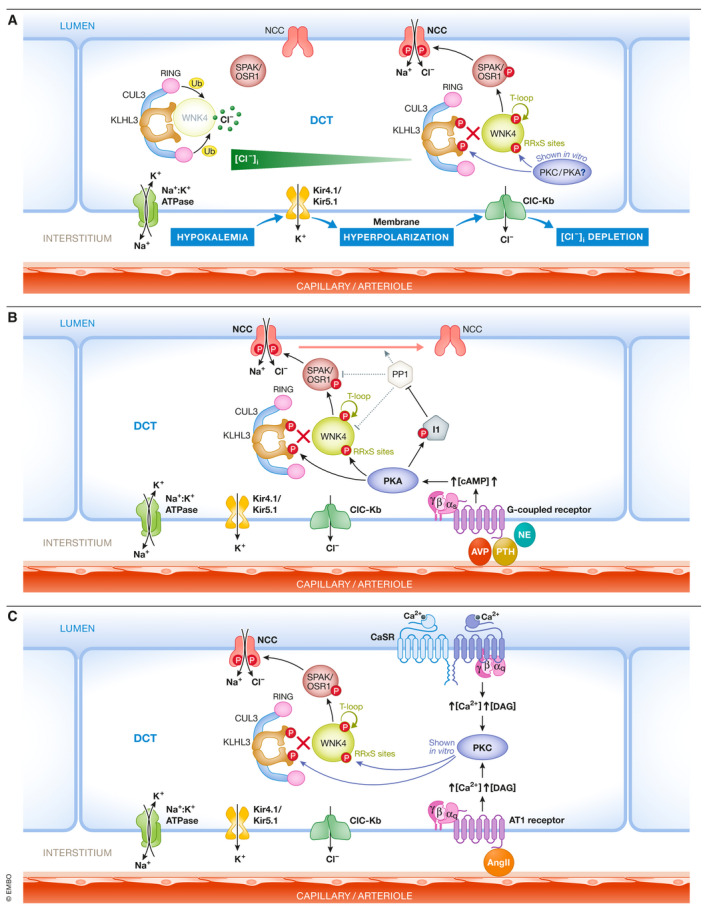
Signal transduction systems that mediate NCC regulation in response to extracellular stimuli (A) DCT cells directly sense changes in [K^+^]_e_ that modulate NCC activity through modulation of the [Cl^‐^]_i_ that in turns modulates WNK4‐SPAK activity. (B) Hormonal stimuli that activate Gαs‐coupled receptors and promote PKA activation in the DCT lead to NCC activation through different pathways activating WNK4 and inhibiting its KLHL3/CUL3 E3‐mediated degradation. In addition, PKA phosphorylate I1, a PP1 regulatory protein whose expression is enriched in DCT cells. I1 phosphorylation enhances its ability to inhibit PP1 and thus may prevent PP1‐mediated dephosphorylation of WNK4, SPAK/OSR1, and NCC. (C) The Ca^2+^‐sensing receptor (CaSR) and the ATI angiotensin II receptor both activate Gαq proteins that promote activation of PKC isoforms. In vitro, PKC can phosphorylate WNK4 and KLHL3 RRxS sites leading to increased WNK4 protein and activity levels. In vivo, volume depletion or increased Ca^2+^ delivery to the DCT, which would be expected to promote ATI and CaSR respectively, correlate with increased phosphorylation of these sites. Created with Biorender.com.

Regarding the molecular mechanisms involved, the currently accepted model is that, through Kir4.1/5.1 K^+^ channels, DCT cells directly sense changes in [K^+^]_e_ and modulate the levels of NCC activity accordingly (Fig 3A). Alterations in [K^+^]_e_ affect movement of K^+^ ions across DCT’s basolateral membrane, leading to changes in basolateral membrane potential that ultimately affect the driving force for Cl^−^ ion movement through basolateral ClC‐Kb channels. Additionally, low dietary potassium intake increases, and high dietary potassium intake decreases, the conductance of Kir4.1/5.1 channels, amplifying the effects of driving force on basolateral voltage (Wang *et al*, 2018). Thus, changes in [Cl^−^]_i_ occur that affect the activity of the WNK4‐SPAK/OSR1 pathway through direct modulation of WNK4 activity. For instance, when [K^+^]_e_ decreases, net K^+^ efflux through basolateral K^+^ channels in the DCT promotes membrane hyperpolarization that drives Cl^−^ efflux. The resulting decrease in [Cl^−^]_i_ stimulates WNK4 activity leading to NCC activation. The opposite mechanism operates in the face of hyperkalemia.

Supporting this model and consistent with the role of Kir4.1/5.1 heterotetramers in the regulation of NCC activity, it has been shown that, conditional kidney‐specific Kir4.1 knockout mice have decreased activity, expression and phosphorylation of NCC, lower DCT basolateral K^+^ conductance, depolarized basolateral membranes, and a Gitelman‐like phenotype (Cuevas *et al*, 2017). These observations clearly indicate that Kir5.1 alone cannot form functional K^+^ channels in the DCT, as supported by observations made in vitro (Tucker *et al*, 1996; Tanemoto *et al*, 2005). In contrast, Kir5.1 knockout mice display higher activity, expression, and phosphorylation of NCC, higher DCT basolateral K^+^ conductance, and hyperpolarized basolateral membranes (Paulais *et al*, 2011; Wu *et al*, 2019). Thus, it appears that Kir4.1 can itself form functional homomeric potassium channels in the absence of Kir5.1. It has been proposed that decreased sensitivity to intracellular pH of Kir4.1 homotetramers (versus Kir4.1/5.1 heterotetramers) may explain the higher conductance mediated by Kir4.1 homotetramers in the DCTs of these mice.

Supporting the role of [Cl^−^]_i_ on NCC modulation during changes in dietary K^+^, it was observed that Cl^−^ channel activity and Cl^−^ conductance of DCT cells are increased in animals on low K^+^ diet and decreased on high K^+^ diet (Cuevas *et al*, 2017). These effects were abrogated in Kir4.1 knockout mice. In addition, Barttin hypomorphic mice (Barttin is a chaperone protein that is required for ClC‐Kb channel localization in the plasma membrane) did not upregulate pNCC levels when placed on low K^+^ diet (Nomura *et al*, 2018). Finally, mice harboring mutations in the WNK4 gene that make the kinase insensitive to inhibition by Cl^−^ (WNK4‐L319,321F mice) displayed higher basal levels of NCC and pNCC, an FHHt‐like phenotype, and impaired regulation of NCC in response to changes in [K^+^]_e_ (Chen *et al*, 2019).

Experimental evidence suggests that the “turning off” of the WNK4‐SPAK/OSR1 kinase cascade due to increased [Cl^−^]_i_ contributes to the dephosphorylation of NCC in response to high K^+^ intake and hyperkalemia (Chen *et al*, 2019; Mukherjee *et al*, 2021). Additionally, there is evidence that activation of phosphatases is also important to dephosphorylate and thus decrease NCC activity (Penton *et al*, 2016; Chen *et al*, 2019). Phosphatases that have been suggested to participate in NCC dephosphorylation include PP1 (Penton *et al*, 2019), PP3 (or calcineurin) (Hoorn *et al*, 2011; Lazelle *et al*, 2015), and PP4 (Glover *et al*, 2010). Of note, tacrolimus is a calcineurin‐specific inhibitor that is widely used as an immunosuppressor in transplant recipients and for the treatment of autoimmune disease, and its use has been associated with the development of hyperkalemic hypertension that correlates with NCC activation (Hoorn *et al*, 2011; Rojas‐Vega *et al*, 2015a).

In experiments performed with kidney slices incubated ex vivo with solutions containing different K^+^ concentrations, none of the inhibitors directed against any of the mentioned phosphatases was able to prevent the dephosphorylation of NCC stimulated by high [K^+^]_e_ (Penton *et al*, 2016; Mukherjee *et al*, 2021). However, pre‐treatment with tacrolimus was shown to attenuate NCC dephosphorylation in mouse kidneys in response to acute oral K^+^ loading (15 min post‐gavage) (Shoda *et al*, 2017). Further research will be necessary to clarify the source of these controversial observations and to understand the role of each specific phosphatase in this pathway.

In addition to the modulation of WNK4 kinase activity by Cl^−^ binding, another mechanism for NCC modulation in response to changes in [K^+^]_e_ involves modulation of WNK4 expression levels in the DCT. Mice placed on low K^+^ diet present higher levels of KLHL3 phosphorylation at a site located within the substrate binding domain (Ishizawa *et al*, 2016) that prevents substrate binding and thus, KLHL3‐targeted WNK4 degradation (Shibata *et al*, 2014). Thus, mice on low K^+^ diet display higher levels of WNK4 in the kidney. The molecular mechanisms leading to this phosphorylation event remain elusive, although it has been proposed that activation of a PKC isoform may be involved (Ishizawa *et al*, 2016).

It is worth mentioning here that, once this modulation of NCC activity by [K^+^]_e_ was well established and the molecular mechanisms for this modulation began to be uncovered, previous observations that suggested that NCC is a target for modulation by the mineralocorticoid hormone aldosterone were immediately questioned. In these works, it was observed that aldosterone injection or infusion in rodents promoted an increase in NCC expression levels and phosphorylation (Kim *et al*, 1998; van der Lubbe *et al*, 2011, 2012). Aldosterone is synthesized and secreted in the zona glomerulosa of the adrenal gland in response to hypovolemia (angiotensin II) and hyperkalemia. It acts upon cells of the CNT and principal cells of the CD to promote Na^+^ reabsorption and K^+^ secretion. This is achieved through the stimulation of the activity of the apical Na^+^ channel ENaC and of the basolateral Na^+^/K^+^ ATPase, whose activity indirectly promotes K^+^ efflux through the apical ROMK K^+^ channels (Fig 1B). Thus, one of the consequences of increased circulating aldosterone in the absence of high K^+^ intake or hyperkalemia is hypokalemia, which would be expected to directly activate NCC. To evaluate whether aldosterone can activate NCC directly or only indirectly through its effects on [K^+^]_e_, clever experimental strategies were designed by different groups. Czogalla *et al* showed that in mice in which the mineralocorticoid receptor (MR) was randomly deleted in ~20% of renal tubule cells, pNCC upregulation in response to low NaCl diet was similar in cells expressing than in cells not expressing MR, suggesting that the effect was independent of MR activity (Czogalla *et al*, 2016). In contrast, upregulation of αENaC abundance with low Na^+^ diet was readily observed in MR positive cells, but was absent in MR negative cells. In another work, Terker *et al* generated kidney‐specific MR knockout mice and observed that these mice had hyperkalemia and reduced NCC phosphorylation levels (Terker *et al*, 2016a). However, pNCC levels could be reverted to those observed in wild‐type littermates by administration of a low K^+^ diet, suggesting that altered pNCC levels were secondary to the hyperkalemia observed in the knockouts.

In summary, NCC activity is tightly regulated in response to changes in [K^+^]_e_ and the mechanisms underlying this regulation are now partially understood, although research is still underway to further increase our understanding. NCC is not a direct target of aldosterone‐mediated regulation, although aldosterone can affect NCC activity indirectly by affecting [K^+^]_e_. Given that NCC is an important regulator of renal Na^+^ excretion, modulation of NCC activity by [K^+^]_e_ may at least partially explain the inverse correlation between K^+^ consumption and blood pressure levels observed at the population level in humans and in diverse animal models. Supporting this concept, it has been shown that the increase in blood pressure produced in wild‐type mice given a high Na^+^/low K^+^ diet is not observed in NCC knockout mouse (Terker *et al*, 2015b). Other molecular pathways explaining the effect of K^+^ consumption on blood pressure may include additional effects of K^+^ on renal cotransporters (Murillo‐de‐Ozores *et al*, 2018) or extra‐renal mechanisms involving, for example, the vasculature and central nervous system (Adrogue & Madias, 2007).

## Additional mechanisms implicated in the modulation of NCC activity

In addition to [K^+^]_e_, NCC activity is modulated by multiple hormonal stimuli (Rojas‐Vega & Gamba, 2016). Hormones that increase the activity of NCC include angiotensin II (angII), arginine vasopressin (AVP), insulin, norepinephrine (NE), parathyroid hormone (PTH), and female sex hormones (Pedersen *et al*, 2010; van der Lubbe *et al*, 2011; Castaneda‐Bueno *et al*, 2012; Chavez‐Canales *et al*, 2013; Rojas‐Vega *et al*, 2015b). Hormonal binding to their respective transmembrane receptors promotes activation of signaling cascades that, in some cases, share downstream components. For example, AVP, NE, and PTH are known to act on Gαs‐coupled receptors to increase the cAMP levels in the cell, activating the Protein Kinase A (PKA). In kidney slices, pharmacological modulation of cAMP levels can indeed modulate NCC phosphorylation (Penton *et al*, 2019). Three different pathways have been proposed to mediate NCC activation by PKA (Fig 3B). PKA can phosphorylate KLHL3 and WNK4 in RRXS motifs in *in vitro* systems (Yoshizaki *et al*, 2015; Castaneda‐Bueno *et al*, 2017). The RRXS motif in KLHL3 lies within the substrate binding domain. Phosphorylation of this site (serine 433) affects KLHL3 binding to WNK kinases, preventing their degradation. In addition, phosphorylation of WNK4 at RRXS sites promotes the kinase’s activation (Castaneda‐Bueno *et al*, 2017). The third pathway involves phosphorylation by PKA of the Protein Phosphatase 1 (PP1) regulatory protein Inhibitor 1 (I‐1). Phosphorylation of I‐1 by PKA activate I‐1 to inhibit PP1. I‐1 expression is enriched in DCT cells in the kidney and I‐1 knockout mice have reduced pNCC levels, as well as reduced response to PTH and NE in terms of NCC activation (Penton *et al*, 2019). PP1 modulates pNCC, pSPAK, and pWNK4 levels (Murillo‐de‐Ozores *et al*, 2018; Penton *et al*, 2019), and thus, modulation of PP1 activity may affect the pathway at these three levels.

Angiotensin II and extracellular Ca^2+^ ions activate the Gαq‐coupled AT1 and CaSR receptors, respectively. Gαq activation promotes activation of Protein Kinase C (PKC). In vitro assays have shown that some PKC isoforms are also able to phosphorylate KLHL3 and WNK4 at RRXS motifs, exerting similar effects on these proteins as the ones described above for PKA (Shibata *et al*, 2014; Castaneda‐Bueno *et al*, 2017; Bazua‐Valenti *et al*, 2018) (Fig 3C). In in vitro and in vivo systems, these hormones have been shown to promote phosphorylation of both KLHL3 and WNK4 (Shibata *et al*, 2014; Castaneda‐Bueno *et al*, 2017; Bazua‐Valenti *et al*, 2018). Finally, AKT‐mediated phosphorylation of KLHL3 at the RRXS motif has also been shown to occur in vitro, suggesting that this pathway could also participate in insulin‐mediated NCC activation (Chavez‐Canales *et al*, 2013; Yoshizaki *et al*, 2015).

## The KS‐WNK1 enigma

As explained above, a short isoform of WNK1 that lacks the catalytic domain is expressed exclusively in the kidney, hence its name: Kidney‐Specific WNK1. Data from qPCR assays and RNA sequencing of dissected tubule segments have shown that its expression is restricted to the DCT, where mRNA levels are very high, and to the connecting tubule, where mRNA levels are lower (Vidal‐Petiot *et al*, 2012; Chen *et al*, 2021a).

Despite the lack of a kinase domain, in X. laevis oocytes KS‐WNK1 expression promotes NCC activation. This effect depends on KS‐WNK1 interaction with a full‐length WNK kinase (in this case, the endogenous WNK expressed by the oocytes) because it is prevented by addition of the highly specific WNK inhibitor WNK463 or if the C‐terminal coiled‐coil domain involved in heterodimerization is mutated to prevent WNK‐WNK binding (Argaiz *et al*, 2018). In addition, when coexpressed with WNK4, KS‐WNK1 promotes its activating phosphorylation. Thus, in the DCT the KS‐WNK1‐WNK4 interaction may be important to achieve full activation of WNK4 under certain conditions. These results agree with in vivo observations showing that in situations in which NCC is activated, like for instance, in mice fed with low K^+^ diet or in KLHL3‐R528H mice, KS‐WNK1, WNK4, and SPAK form intracytoplasmic aggregates known as WNK bodies. Formation of these structures requires the presence of KS‐WNK1, since they are not observed in KS‐WNK1 knockout mice under similar conditions (Boyd‐Shiwarski *et al*, 2018; Thomson *et al*, 2020) (Ostrosky‐Frid *et al*, 2021).

In vivo evidence of a positive role of KS‐WNK1 on NCC activity was recently provided with the description of mutations in the region encoding the acidic domain of WNK1 that cause a mild FHHt phenotype in humans (hyperkalemic metabolic acidosis) (Louis‐Dit‐Picard *et al*, 2020). No hypertension was observed in these patients. However, a mouse model carrying a deletion of one amino acid residue of the acidic domain of WNK1 provided some data indicative of a mild volume expansion phenotype (lower renal renin mRNA levels). Of note, these mice presented higher levels of SPAK, pSPAK/OSR1, NCC, and pNCC, as well as a similar clinical phenotype to the patients. The mutation of the acidic domain is expected to produce an increase in expression of the protein, as the acidic domain is the binding site for the KLHL3‐CUL3 E3 complex that regulates WNK degradation. In vitro data provided in the same study confirmed that KS‐WNK1 is highly sensitive to KLHL3‐targeted degradation, which is prevented in the presence of the acidic domain mutations. Interestingly, however, the long form of WNK1 (L‐WNK1) was much less sensitive to KLHL3‐targeted degradation. This, together with the evidence explained in a previous section suggesting that L‐WNK1 is not expressed in the DCT, supports the notion that KS‐WNK1 overexpression in these patients and mice is responsible for the mild FHHt phenotype.

Nevertheless, the role of KS‐WNK1 in DCT physiology is still controversial, and this is mainly derived from the phenotype observed in KS‐WNK1 knockout mice. A few years back, Hadchouel *et al* and Liu *et al* generated KS‐WNK1 knockout models that presented increased pNCC and NCC levels (Hadchouel *et al*, 2010; Liu *et al*, 2011). These observations were originally well accepted given that initial in vitro experiments performed with KS‐WNK1 had suggested that KS‐WNK1 exerted a negative role on NCC activity (Subramanya *et al*, 2006). However, despite the higher pNCC levels, no FHHt‐like electrolytic alterations were observed. It has been suggested that activation of NCC in these models could be secondary (Ostrosky‐Frid *et al*, 2019); that is, given that recent studies suggest a role for KS‐WNK1 in the response to hypokalemia (Boyd‐Shiwarski *et al*, 2018), it is possible that KS‐WNK1 knockout mice have a potassium losing phenotype that could be compensated by a WNK4‐induced activation of NCC.

The above‐mentioned role of KS‐WNK1 on NCC regulation in response to decreases in [K^+^]_e_ agrees with the recent observation that levels of KS‐WNK1 protein are greatly upregulated in mice on low K^+^ diet (Ostrosky‐Frid *et al*, 2021). Interestingly, KS‐WNK1 protein levels were undetectable in blots of mice on normal diet, but were high in KLHL3‐R528H animals, suggesting that the low levels in wild‐type mice on normal chow may be due to a high rate of KLHL3‐targeted degradation, and that upregulation in the low K^+^ diet may be due to decreased activity of the KLHL3‐CUL3 E3 ubiquitin ligase (Ishizawa *et al*, 2016). Thus, KS‐WNK1 is highly sensitive to the KLHL3‐CUL3 E3 ubiquitin ligase complex that in mice on normal chow is active, complicating the understanding of the physiological role of this variant.

## Concluding remarks

The discovery of genes involved in the regulation of DCT function opened the possibility to elucidate, at the molecular level, a complex network of transporters, kinases, ubiquitin ligases, and protein phosphatases involved in the modulation of renal salt reabsorption in this nephron segment that defines blood pressure levels and K^+^ homeostasis. The description of the indirect effect that NCC activity exerts on renal K^+^ secretion and the characterization of the molecular pathways that mediate NCC regulation in response to changes in [K^+^]_e_ have promoted much recent interest among researchers in the field, as it has been proposed that this pathway may be key to explain, at least partly, the effect of dietary K^+^ consumption on blood pressure levels in the population. Despite significant advances made, many questions remain unanswered. For instance, FHHt‐causative mutations in WNK4 or in the KLHL3‐CUL3 E3 complex result in increased expression of WNK4. However, mechanisms have been described that would be expected to turn off the kinase under conditions of volume expansion and hyperkalemia. Thus, it is unclear why these mechanisms do not compensate for the increase in kinase’s expression. In this regard, in heterologous expression systems, exogenously expressed WNK4 is inhibited under high [Cl^−^]_i_ and activity /phosphorylation of downstream components cannot be restored by increasing the amount of WNK4 expressed. This suggests that mutations in WNK4 or in KLHL3/CUL3 complex proteins may also alter the modulation of WNK4 by intracellular chloride. The role of the KS‐WNK1 isoform in this setting may be critical since apparently the presence of KS‐WNK1 modulates WNK4’s sensibility to chloride. It is much what we have learned in the past decade about molecular mechanism of hypertension; however, there is still much more to explore. As the late Gerhard Giebisch, one of the greatest mentors for all renal physiologists, used to say: “we remain confused, but confused on a higher level”.

## Author contributions

MC‐B, DHE, and GG contributed to the conception, the writing, and the editing of the manuscript.

## Conflict of interest

The authors declare that they have no conflict of interest.

## For more information

- Gitelman’s syndrome OMIM entry: https://www.omim.org/entry/263800
- FHHt type A OMIM entry: https://www.omim.org/entry/145260
- FHHt type B OMIM entry: https://www.omim.org/entry/614491
- FHHt type C OMIM entry: https://www.omim.org/entry/614492
- FHHt type D OMIM entry: https://www.omim.org/entry/614495
- FHHt type E OMIM entry: https://www.omim.org/entry/614496
- Bartter syndrome type 3 OMIM entry: https://www.omim.org/entry/607364
- SESAME/EAST syndrome OMIM entry: https://www.omim.org/entry/612780
- Author’s website: https://www.biomedicas.unam.mx/personal‐academico/gerardo‐gamba‐ayala/
- Author’s website: https://www.incmnsz.mx/Investigacion/investigador.jsp?id=3
- Author’s website: https://www.ohsu.edu/providers/david‐h‐ellison‐md

**Pending issues**

- Understanding the mechanisms that maintain mutant WNK4 active in FHHt, despite the electrolytic alterations and increased blood pressure that would be expected to turn off the pathway.
- Elucidating the physiological role of KS‐WNK1.
- Uncovering the kinases responsible for the in vivo phosphorylation of RRXS motifs in WNK4 and KLHL3 in response to hypokalemia and volume depletion.
- Uncovering the molecular mechanisms that lead to distal nephron remodeling in response to changes in K^+^ intake and in response to alterations in NCC function.
- Understanding the molecular mechanism of FHHt in patients with the missense mutations R1185C and K1169E in WNK4 that lie within the C‐terminal domain of the protein.
